# Access to HIV treatment and care in Armenia

**DOI:** 10.1186/1742-4690-9-S1-P75

**Published:** 2012-05-25

**Authors:** S Grigoryan, A Mkrtchyan

**Affiliations:** 1 National Center for Aids Prevention, Yerevan, Armenia

## Introduction

ART has become available in Armenia since 2005. To evaluate progress in the response of the National Programme to PLHIV’s needs since last evaluation time, UNAIDS country office requested WHO/Europe HIV/AIDS treatment and care expert for a country mission. The purpose of the mission was to evaluate access of PLHIV to HIV/AIDS treatment and care and other related services.

## Materials and methods

Prior to the mission some background documents had been reviewed. During the mission key-informant interviews were conducted with a range of stakeholders. Also, a focus group discussion was conducted with PLHIV representing two NGOs. Visits were made to a number of institutions and activities in Yerevan, the capital.

## Results

As of end 2010, cumulative 971 HIV cases were registered by the National Center for AIDS Prevention (NCAP), 231 persons died. Of 740 PLHIV 542 (73%) were seen for HIV care in 2010. Armenia has accepted a higher threshold of ART initiation (CD43), as recommended by recent WHO guidelines. There is no waiting list for starting ART. By the end 2010, 250 PLHIV (46% of seen for care) were on ART. Adherence to ART is pretty high. Of all 294 PLHIV ever started ART, only 44 (14.9%) dropped it out. However, more than a half of them (25) died, due to late presentation. About a quarter of newly identified HIV patients had CD43at the time of diagnosis.

## Conclusions

NCAP has demonstrated high coverage with HIV care and ART. PLHIV are satisfied with the range and quality of services they receive. However, low level of CD4 cell count in more than a half newly diagnosed HIV cases (57.7% PLHIV had CD4 <350cells/mm^3^ at time of diagnosis) in combination with the highest proportion of PLHIV identified due to clinical symptoms compared to other reasons for HIV testing (39.8%), indicates late presentation and raises concern of the health system’s inefficiency to diagnose HIV at early stages and thus to early enroll in HIV care. The expert has made appropriate recommendations to improve early HIV detection for optimal care and patients’ survival.

